# U.S. Food and Drug Administration’s Patient-Focused Drug Development Initiative: Experience with Integration of Patient-Experience Data in a New Drug Application for Esketamine Nasal Spray Plus a Newly Initiated Oral Antidepressant for Treatment-Resistant Depression

**DOI:** 10.1007/s43441-021-00340-6

**Published:** 2021-10-08

**Authors:** Eva G. Katz, Pauline McNulty, Bennett Levitan, Patricia Treichler, Jadwiga Martynowicz, Carol Jamieson

**Affiliations:** 1 Janssen Research & Development, LLC, 700 U.S. Highway 202 South, Raritan, NJ 08869 USA; 2 Janssen Research & Development, LLC, Titusville, NJ USA; 3 Janssen Research & Development, LLC, Milpitas, CA USA

**Keywords:** Patient-Focused Drug Development, Patient-experience, Patient preference, Patient-reported outcomes, Esketamine nasal spray, Treatment-resistant depression

## Abstract

The Patient-Focused Drug Development initiative of the U.S. Food and Drug Administration (FDA) aims to ensure that the patient experience of disease and treatment is an integral component of the drug development process. The 21st Century Cures Act and Prescription Drug User Fee Act (PDUFA) VI require the FDA to publicly report the type of patient-experience data reviewed in a new drug application (NDA) to inform regulatory decision-making. This report describes a recent approach adopted at Janssen of integrating patient-experience data into the NDA for esketamine (SPRAVATO®) nasal spray with a newly initiated oral antidepressant (esketamine + AD) for treatment-resistant depression. During the development of esketamine + AD, patient-experience data were collected using several patient-reported outcomes, including the Sheehan Disability Scale and 9-item Patient Health Questionnaire (PHQ-9). Additionally, a patient-preference study assessed the relative importance of benefits and harms that patients allocated to different attributes of treatment. Preferences were collected from patients enrolled in phase 3 esketamine trials and from an online panel of primarily ketamine-naive patients. Patient-experience data were integrated into the esketamine NDA, the FDA advisory committee meeting briefing document, and the Sponsor’s presentation. The FDA acknowledged reviewing the patient-experience data and determined that they supported esketamine + AD for treatment-resistant depression. This report highlights the importance of integrating patient-experience methods early in drug development, their impact on assessing patient-relevant benefits and risks, and how they can help improve clinical program design.

## Introduction

The Patient-Focused Drug Development (PFDD) initiative of the U.S. Food and Drug Administration (FDA) has evolved to ensure that the patient-experience of disease and treatment becomes an integral component of drug development and regulatory review [1]. The FDA encourages collecting patient-experience data in clinical trials to capture and support the patient perspective as a part of the regulatory filing. Furthermore, the 21st Century Cures Act (Section 3001 [b]) and Prescription Drug User Fee Act (PDUFA) VI require the FDA to publicly report the type of patient-experience data that are reviewed in a new drug application (NDA) to inform regulatory decision-making [2, 3]. Consequently, as part of its review documentation, the FDA now posts a patient-experience data checklist that explicitly summarizes the types of patient-experience data included in the NDA. In July 2020, the FDA added the checklist to the electronic common technical document, allowing sponsors to complete the checklist and include it in the Reviewer’s Guide [4]. Under these Acts, the FDA is also required to issue PFDD guidance documents in the ensuing 5 years detailing methods and approaches to collecting patient-experience data, and how such data and any related information can be used in the drug development process. The first such guidance, with information on how to determine the target patient population for collecting patient-experience data and which sampling strategies may be appropriate for addressing potential research question(s), was published in June 2020 [5].

The FDA considers patient-experience data to include information from patients about (1) the signs and symptoms of their condition and how they affect patients’ day-to-day functioning and quality of life, (2) the natural history of their condition and the changes in symptoms over time, (3) their experience with the symptoms and burdens related to treatment, (4) their views on potential disease or treatment outcomes and how they weigh the importance of different possible outcomes, and (5) how they view the impact of the disease, treatment, and outcomes, and potential tradeoffs between disease outcomes and treatment benefits and risks [5]. These patient-experiences are becoming increasingly important in the development and regulatory review of new therapies [6, 7].

Esketamine (SPRAVATO®), the S-enantiomer of ketamine, was developed by Janssen and is now approved in the US (since March 5, 2019) [8], Europe, and numerous other countries for use as a nasal spray in conjunction with a newly initiated oral antidepressant (esketamine + AD) for the treatment of adults with treatment-resistant depression (TRD) [9]. Collecting patient-experience data and including them in the NDA for regulatory approval of a new treatment is particularly important for conditions such as depression, where objective scales to assess prognosis are lacking. To capture patient perspectives on functioning, symptoms, benefits, and risks associated with esketamine + AD treatment, Janssen proactively used several methods to collect patient-experience data during the drug development, including a variety of patient-reported outcomes (PROs) and a patient-preference study. These data were included in the esketamine NDA (single application), the FDA briefing information for the Advisory Committee meeting [10], and the Sponsor’s presentation at the Advisory Committee meeting [11].

This report summarizes patient-experience activities assessed in the esketamine clinical development program and the corresponding comments from the FDA. Additionally, it presents a recent approach adopted at Janssen to generating and integrating patient-experience data into the esketamine NDA, as well as the FDA regulatory review and decision-making process.

## Phase 3 Clinical Trials of Esketamine: TRANSFORM and SUSTAIN

The efficacy and safety of esketamine + AD was assessed in international, phase 3 TRANSFORM and SUSTAIN trials in adults with TRD. The TRANSFORM trials were randomized, double-blind, multicenter, active-controlled (patients received a newly initiated AD plus intranasal placebo), phase 3 studies that assessed the efficacy, safety, and tolerability of esketamine + AD. TRANSFORM-1 (NCT02417064) and TRANSFORM-2 (NCT02418585) assessed fixed and flexible doses, respectively, of esketamine + AD [12, 13]. SUSTAIN-1 (NCT02493868) was a double-blind, multicenter, randomized withdrawal, phase 3 trial that assessed the efficacy of esketamine + AD in delaying relapse of depressive symptoms after induction and optimization [14]. SUSTAIN-2 (NCT02497287) was an open-label phase 3 trial that assessed long-term safety and efficacy of esketamine + AD in patients with TRD [15]. SUSTAIN-3 (NCT02782104) is an ongoing open-label phase 3 trial that enrolled patients from short-term esketamine clinical trials to receive long-term treatment with esketamine. The study protocols and statistical analysis plans are available in public domain for TRANSFORM-1 [16, 17], TRANSFORM-2 [18, 19], SUSTAIN-1 [20, 21], and SUSTAIN-2 [22, 23]. Data from all TRANSFORM and SUSTAIN trials were included in the esketamine NDA [24].

## Patient-Reported Outcomes

The PRO strategy included research to understand which symptoms and impacts of depression are important to patients and how they perceive effects of depression and its treatment on health and functioning (literature review not available in the public domain). Specific PROs were carefully selected, focusing on key concepts of the patients experience of depression, with the consideration that patient “feelings and function” are adequately captured. Evidence supporting the content validity and measurement properties of the PROs was reviewed to assess whether the instruments were fit for purpose in the context of the phase 3 trials. When gaps in the supportive evidence were identified, additional data (patient interviews to confirm content validity, psychometric evaluation of measurement properties, and Electronic Clinical Outcome Assessment [eCOA] usability study that were submitted to the FDA but are not in the public domain) were generated to support selected PROs for use in the phase 3 TRD trials. Phase 2 clinical trials included limited PRO assessments that assisted with selection of PROs, and in addition, patients receiving investigational drugs for TRD in two phase 2 trials were interviewed to assess their experience of treatment, including positive and negative health effects [25]. Results from these interviews further helped to confirm the relevant concepts identified for assessment in phase 3 clinical trials. Feedback on the proposed PROs was solicited from FDA and European Medicines Agency.

The 9-item Patient Health Questionnaire (PHQ-9), a PRO that includes items addressing the Diagnostic and Statistical Manual of Mental Disorders (5th edition) criteria for depression, was selected to assess patient perceptions of symptoms of depression. Changes in social, family, and work functioning were identified as important functional impacts of depression that are not adequately captured by the primary endpoint measure for the clinical trials, ie, change in the Montgomery-Åsberg Depression Rating Scale (MADRS) from baseline to day 28 [12, 13]. The Sheehan Disability Scale (SDS), a PRO commonly used to address functional impairment in depression trials, was selected to assess functioning. Two additional PROs, EQ-5D-5L and 7-item Generalized Anxiety Disorder (GAD-7) scale, were used. The EQ-5D-5L instrument captured broader concepts of mobility, self-care, usual activities, pain/discomfort, anxiety/depression, and overall health. The GAD-7 scale evaluated comorbid anxiety.

The SDS and PHQ-9 PROs, which are validated instruments that are widely used to assess experience of symptoms and disability in patients with depression [26–28], were included as key secondary endpoints in TRANSFORM-1 and TRANSFORM-2 after consultation with the FDA. The primary and 3 key secondary endpoints were tested in the following order: change in MADRS total score (primary endpoint), change in clinical response by day 2, change in SDS total score, and change in PHQ-9 total score. For TRANSFORM-2, an endpoint was considered statistically significant if the endpoint was individually significant and all previous endpoints in the hierarchy were statistically significant at a prespecified level. For TRANSFORM-1, since 2 doses were tested, a truncated fixed sequence procedure was used, where testing proceeded to the next endpoint in the hierarchy only if at least 1 dose-to-placebo comparison was significant at a prespecified level. The key secondary PRO endpoints were included in the fixed sequence approach to adjust for multiplicity and to strongly control type I error across the primary and the three key secondary efficacy endpoints as these key secondary endpoint were intended to be included in the product label.

The same PROs were also included as secondary endpoints in SUSTAIN-1 and SUSTAIN-2. All 4 trials included GAD-7 and EQ-5D-5L as secondary endpoints. The FDA’s PRO guidance was followed regarding appropriate implementation of PROs in clinical trials [29].

For the PRO data from the TRANSFORM clinical trials, change from baseline in instrument scores was assessed at day 28 during the double-blind induction phase. The statistical plan in these trials required the PHQ-9 and SDS scores to be assessed in a hierarchical manner after another secondary endpoint of clinical response at day 2 that was maintained through day 28. Additional, PRO analyses were performed in the clinical trials but are not reported here.

**Fig. 1:**
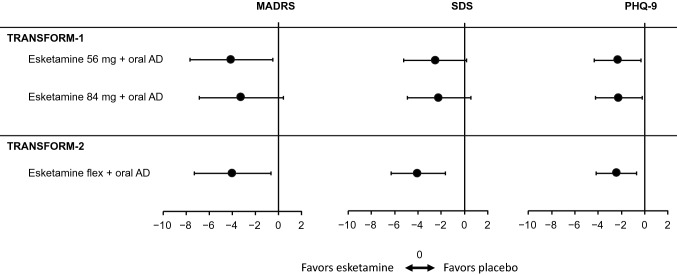
Least squares mean difference (± 95% CI) in change from baseline in primary and key PRO secondary endpoints at day 28: data from TRANSFORM-1 and TRANSFORM-2. *AD* antidepressant, *flex* flexible dose, *MADRS* Montgomery-Åsberg Depression Rating Scale, *PHQ-9* 9-item Patient Health Questionnaire, *PRO* patient-reported outcome, *SDS* Sheehan Disability Scale

In both TRANSFORM-1 and TRANSFORM-2, the PRO results were consistent with and supportive of the primary endpoint (ie, change in the MADRS from baseline to day 28) [12, 13]. The least squares mean (LSM) difference in change from baseline in MADRS, SDS and PHQ-9 scores at day 28 numerically favored esketamine + AD compared with AD + placebo (Fig. 1). In TRANSFORM-1, the LSM (95% CI) difference in change from baseline to day 28 in SDS scores was − 2.5 (− 5.25 to 0.2) with esketamine 56 mg plus AD and − 2.2 (− 4.91 to 0.53) with esketamine 84 mg plus AD; the corresponding PHQ-9 scores were − 2.3 (− 4.34 to − 0.31) and − 2.2 (− 4.26 to − 0.20), respectively [12]. Similarly, in TRANSFORM-2, the LSM (95% CI) difference in change from baseline to day 28 in SDS scores was − 4.0 (− 6.28 to − 1.61) with esketamine flexible dose (56 or 84 mg) plus AD; the corresponding PHQ-9 scores were − 2.4 (− 4.18 to − 0.69) [13]. In both trials, the PROs were not formally analyzed for statistical significance because of the prespecified hierarchical design that required one or more endpoints earlier in the hierarchical order to be statistically significant.

## Patient-Preference Study

A discrete-choice survey was conducted to evaluate relative importance of benefits and harms associated with treatment and to quantify the maximum acceptable risk patients would accept for treatments that relieved depression. Specifically, degree of depression relief (efficacy), time to response, transitory postdose issues—a composite attribute of postdose short-term altered sensations and the inconvenience associated with logistics of drug administration—and putative long-term extreme safety risks associated with ketamine abuse (i.e., cognitive-impairment and ulcerative cystitis) were included in the preference survey. Planning for the preference study began prior to the start of the phase 3 trials. Development, implementation, and analysis of the preference study spanned more than 1.5 years. The comprehensive and detailed patient-preference study design along with the complete statistical information (analysis, methods interpretations, estimation methods, summary results and limitation) and preference study have been published elsewhere [30]. The comprehensive and detailed post-hoc benefit-risk assessment including statistical methods, analysis results, tabular summaries, interpretation of results, and limitations have also been recently published [31].

Because patients with TRD have failed several previous antidepressant therapies, it was unclear whether patients would believe textual descriptions of rapid onset of efficacy without direct experience of novel therapies that provide such efficacy. To address this, the survey was administered to two sets of patients with TRD. The first set was a sample of patients enrolled in SUSTAIN-2 and SUSTAIN-3 at English-speaking sites (USA, Canada, UK, and Australia; *n* = 161) who had direct experience with esketamine, including its efficacy, postdose symptoms, and treatment burden. The second set was a sample of patients from a national online panel (*n* = 301), most of whom had no experience with off-label ketamine; this population represented patients who would consider esketamine + AD, once approved, for the treatment of TRD. Patients in the second set relied solely on textual description of treatments in the survey. Patient preferences between these two sets were compared to inform the degree to which prior experience with esketamine influenced willingness to accept tolerability issues and risks. The survey was completed on a computer or tablet.

In SUSTAIN-2 and SUSTAIN-3, the patient-preference study was a protocol requirement with an independent statistical analysis plan. The data were analyzed using separate models for the clinical trial and the online panel data. These quantitative data provided information on the relative importance of benefits and harms of different treatment attributes and the maximum level of different risks that patients would accept in exchange for different degrees of benefit.

Results from the patient-preference survey demonstrated patient acceptance of the benefit-risk profile of esketamine for TRD [30]. Despite differences in clinical and demographic characteristics, TRD patients in the clinical trial and panel had very similar preferences. Patients valued treatment that would improve mood (from severe to mild symptoms) more than any other feature assessed. Patients placed a low importance on the composite attribute of short-term postdose symptoms and the logistical issues associated with dosing (i.e., 2-h monitoring time and driving restrictions). Interestingly, the relative importance assigned to these attributes by the esketamine-experienced trial sample patients who had directly experienced these challenges was significantly lower than by the primarily (87%) ketamine-naive panel sample patients (*P* = 0.03) [24, 30]. In general, patients with TRD were willing to accept 3% to 5% risk of putative long-term bladder or cognitive-impairment risks in exchange for improvement in symptoms (from severe to moderate) of depression seen with esketamine + AD. Although previously reported as associated with ketamine abuse, such long-term risks were not observed in the esketamine clinical development program and are currently being assessed in SUSTAIN-3.

## Esketamine NDA and FDA’s Review of Patient-Experience Data

**Fig. 2:**
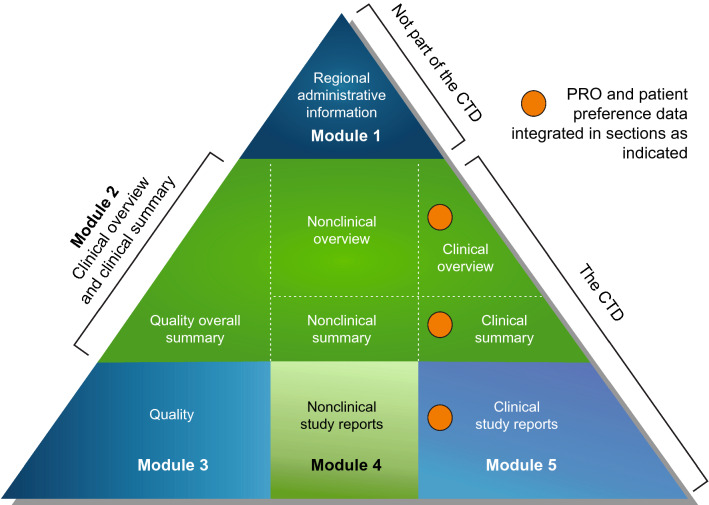
Integration of patient-reported outcomes and patient-preference data into Esketamine NDA submission, shown using CTD triangle. *CTD* common technical document

**Fig. 3:**
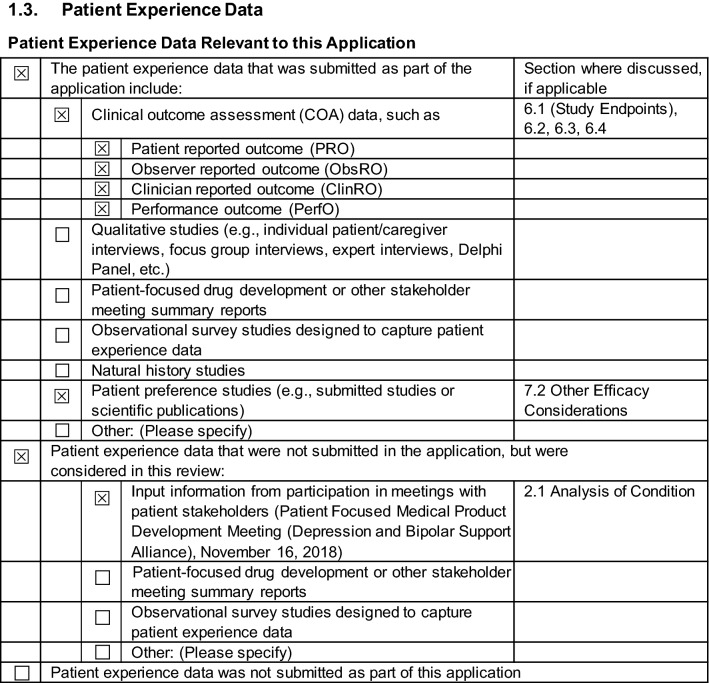
Patient-experience checklist from the FDA’s clinical review of esketamine NDA

Patient-preference and PRO data, which addressed key elements of patient-experience data requested by the FDA, were integrated into the SPRAVATO® NDA as illustrated in the common technical document triangle (Fig. 2) [32]. The PROs were an integral component of the study protocols, statistical analysis plans, and final clinical study reports. The results from PRO and patient-preference assessments were included in the registration package, in the FDA briefing document [10], and at the Sponsor’s presentation at the FDA advisory committee meeting [11]. The FDA reviewed these patient-experience data as indicated in the Patient-Experience Data checklist (Fig. 3) and in several other places in the clinical review document [24]. For PHQ-9, SDS, and EQ-5D-5L PROs, the document noted, “The other secondary outcome measures also corroborated trends towards more improvement in the esketamine arm than the placebo arm, although not within statistically significant ranges” [24]. For the patient-preference study, the document noted, “Overall, these survey results indicate that potential patients with TRD considering esketamine treatment would likely accept the issues with dissociation and waiting time and not driving home in order to obtain clinically significant improvement in their depressive symptoms, but these patients may not be as tolerant of serious issues with cognitive-impairment and bladder toxicity (although these issues were described as “permanent” in the survey which would intensify concern)” [24]. Inclusion of the patient-experience data at the advisory committee meeting presentations by the Sponsor and FDA may have affected the benefit-risk voting for some of the panel members [33]. The FDA used the patient-experience data, including individual testimony, feedback from patient advocacy groups, and functional outcome measures from the clinical trials as part of their assessment to support clinical efficacy of esketamine + AD in TRD [34]. The information and event materials for the FDA advisory committee meeting to discuss efficacy, safety, and risk–benefit profile of esketamine was posted on their web portal in February 2019 [35], and the FDA review of the esketamine NDA was posted on in July 2019, which included the completed patient-experience checklist and extensive discussion on the PRO and preference work [24].

## Discussion

Patient-experience data are considered a valuable part of the totality of evidence evaluated in the regulatory review process and are becoming increasingly important, particularly in the US [6, 7]. These data can profoundly contribute to the full exposition of efficacy and safety of a new treatment and inform clinical trial designs. As in the case of esketamine NDA, the PROs and patient-preferences—two types of patient-experience data—can be effectively used to inform patient-experience and benefit-risk of new treatments. Generating strategies to collect patient-experience data can also provide key supportive information for drug development programs. This experience with esketamine NDA demonstrates the significance of early planning for and integrating patient-experience data in drug development. Although the FDA used the PRO data in its review of esketamine NDA and found them to be supportive of the clinical efficacy data, the SDS and PHQ-9 data were not formally evaluated for statistical significance or included in the esketamine label [9, 12, 13]. This was because although these PROs were prespecified to be assessed in a hierarchical fashion in the statistical analysis plan, one or more endpoints earlier in this hierarchical order were not statistically significant.

The FDA encourages sponsors to discuss patient engagement methods and approaches early in development and often. This is, in part, to ensure that any PRO measure proposed for collecting patient-experience data are clinically relevant and fit for purpose, especially if the PRO data are under consideration for inclusion in the product label. These early discussions may necessitate generation of additional validation data, which could further strengthen the NDA. Importantly, clinical trial sponsors should consider providing a comprehensive package of patient-experience data in the NDA submission so that the regulatory authorities can assess important elements from the patients’ perspective. This could include summary information on PRO findings and data to support validation and interpretation of PROs (where available), as well as patient preferences or other relevant patient-experience data in supporting benefit-risk assessment and overall findings of clinical trial(s). Finally, clinical trial sponsors should consider including information on the PROs in NDA summary documents such as Summary of Clinical Efficacy and Clinical Overview.

## Study Limitations

This was a single example where patient-experience data were integrated into the NDA. More examples are needed to more fully understand the effectiveness of the FDA’s PFDD initiative. A recent study that systematically assessed 59 new drugs approved by the FDA in 2018 found that among 48 approvals that reported whether or not patient-experience data were included in the review, 34 (71%) had included these data [36]. Additionally, the esketamine clinical program did not include all possible methods to capture patient-experience; therefore, we cannot rule out the possibility that some of the patient-experiences with the disease and treatment were not captured or captured accurately. In the patient-preference study, not all patients in the online panel were ketamine-naive; additional limitations of the patient-preference study have been reported previously [30].

## Conclusions

The patient-experience data collected by Janssen and its integration into the NDA for esketamine for the treatment of patients with TRD assisted the FDA in its regulatory evaluation and decision-making. The FDA’s publicly available clinical review of esketamine + AD highlighted its focus on patient-experience data. The FDA acknowledged that functional outcomes were reviewed and provided interpretation on how they supported the primary efficacy endpoint results. Additionally, both the FDA and its advisory committee used the patient-preference data as part of their assessment of patient acceptance of the benefit-risk profile of esketamine + AD. The example of esketamine NDA demonstrates the importance of early planning for and integrating patient-experience methods early in drug development, which can help identify the patient-relevant risks and benefits and ultimately benefit patients and clinical program designs. The data presented here support the FDA’s PFDD initiative and underscore its goal of including patient-experience in its decision-making, especially for conditions such as depression, where objective scales are lacking.
